# *Anncaliia algerae* Microsporidial Myositis, New South Wales, Australia

**DOI:** 10.3201/eid2408.172002

**Published:** 2018-08

**Authors:** Gaurav Sutrave, Adam Maundrell, Caitlin Keighley, Zoe Jennings, Susan Brammah, Min-Xia Wang, Roger Pamphlett, Cameron E. Webb, Damien Stark, Helen Englert, David Gottlieb, Ian Bilmon, Matthew R. Watts

**Affiliations:** Westmead Hospital, Westmead, New South Wales, Australia (G. Sutrave, A. Maundrell, C. Keighley, C.E. Webb, H. Englert, D. Gottlieb, I. Bilmon, M.R. Watts);; University of Sydney, Sydney, New South Wales, Australia (G. Sutrave, M.-X. Wang, R. Pamphlett, C.E. Webb, D. Gottlieb, I. Bilmon, M.R. Watts);; New South Wales Health Pathology Institute of Clinical Pathology and Medical Research, Westmead (Z. Jennings, C.E. Webb, M.R. Watts);; Concord Repatriation General Hospital, Concord West, New South Wales, Australia (S. Brammah);; St. Vincent’s Hospital, Darlinghurst, New South Wales, Australia (D. Stark)

**Keywords:** Anncaliia algerae, microsporidial myositis, New South Wales, Australia, myositis, microsporidia, Anncaliia, infection, immunosuppressed, stem cell transplant, graft versus host disease, parasites, fungi

## Abstract

We describe the successful management of *Anncaliia algerae* microsporidial myositis in a man with graft versus host disease after hemopoietic stem cell transplantation. We also summarize clinical presentation and management approaches and discuss the importance of research into the acquisition of this infection and strategies for prevention.

*Anncaliia algerae* is a microsporidian parasite that infects insects, including mosquitoes, and was first reported as a cause of fatal myositis in 2004 (*1*,*2*). Transmission occurs through contact with spores that are found in water, although the exact mechanism of transmission to humans is unknown (*2*). Myositis has been described in case-patients who were immunosuppressed because of rheumatoid arthritis, solid organ transplantation, and hematologic malignancy (*1*–*5*). It is currently unclear why 4 of the 6 previously published cases have originated in New South Wales, Australia, and the 2 other cases originated in North America (*1*–*5*). We document successful treatment of *A. algerae* infection after hemopoietic stem cell transplantation, provide an update on clinical features and management, and discuss possible routes of transmission and risk-mitigation strategies.

## Case Report

A 66-year-old man sought care at a hospital, reporting a 5-week history of progressive myalgias, fatigue, and weakness. He also had a 3-week episode of nonbloody diarrhea that had resolved a week earlier. He reported no fevers, weight loss, dysphagia, or additional neurologic symptoms. He had chronic graft versus host disease (GVHD) with skin and pulmonary involvement treated with prednisone (25 mg/d orally), methotrexate (15 mg/wk orally), tacrolimus (1 mg 2×/d orally), and fluticasone/salmeterol (250 µg/50 µg 2×/d inhaled). GVHD occurred after a matched unrelated donor, allogeneic bone marrow transplant for acute myeloid leukemia. Before having acute myeloid leukemia, the patient received 6 cycles of combination chemotherapy (rituximab, cyclophosphamide, doxorubicin, vincristine, etoposide, and prednisone) to treat high-grade diffuse large B cell lymphoma. 

The patient lived in a semirural area surrounded by woodland in the Blue Mountains, New South Wales, Australia. His residence had an aboveground molded-plastic rainwater tank that was fed from roof guttering through polyvinyl chloride piping, with an outlet over a mesh-covered opening in the tank cover. Water entering the tank passed through a 5–7-cm layer of decaying plant material and other debris. The tank was periodically used as a source of showering and drinking water.

On examination the patient was afebrile and had exquisite muscle tenderness and edema of the upper and lower limbs. Power was reduced in the upper and lower limb muscles (Medical Research Council grade 3–4 out of 5). Other neurologic findings were unremarkable.

Serum creatine kinase peaked at 858 U/L (reference range 55–150 U/L). On full blood count, hemoglobin was 126 g/L (reference range 130–180 g/L), and lymphocyte count was 0.9 × 10^9^ cells/L (reference range 1.0–4.0 × 10^9^ cells/L). C-reactive protein was 75 mg/L (reference range <3 mg/L), and erythrocyte sedimentation rate was 53 mm/hr (reference range 1–20 mm/hr). Alanine transaminase was 163 U/L and aspartate aminotransferase 235 U/L (reference range <40 U/L for both). Serum albumin nadir was 23 g/L (reference range 35–50 g/L). Serum creatinine, urinary albumin, and urinary protein levels were not elevated. Results of stool microscopy performed using Ryan’s modified trichrome stain were negative for microsporidia.

**Figure 1:**
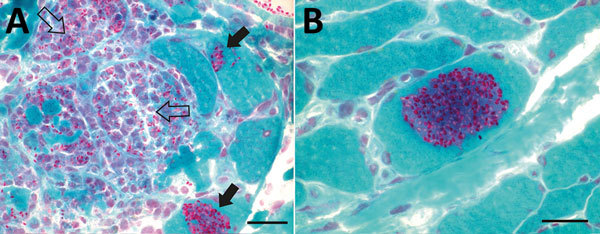
Light micrographs of Gomori trichrome–stained frozen sections of vastus lateralis muscle from a 66-year-old man with *Anncaliia algerae* microsporidial myositis, New South Wales, Australia. A) Necrotising myositis with red-stained, ovoid spores in green-staining viable myocytes (solid arrows) and within macrophages invading necrotic myocytes (open arrows). B) A cluster of red stained, 2–3 µm spores within a viable myocyte. Scale bars indicate 25 µm.

**Figure 2:**
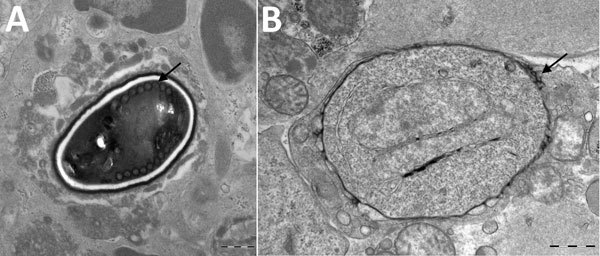
Transmission electron micrographs of vastus lateralis muscle from a 66 year-old man with *Anncaliia algerae* microsporidial myositis, New South Wales, Australia. A) Mature spore with 11 polar tubule coils (arrow) in a single row. Dense exospore and pale endospore. B) Binucleate, proliferative phase meront with characteristic vesicotubular appendages (arrow). Scale bars indicate 500 nm.

Results of nerve conduction studies and electromyography were consistent with myopathy and axonal neuropathy. Magnetic resonance imaging of the lower limbs demonstrated myofascial edema. Light microscopy of a vastus lateralis biopsy demonstrated ovoid organisms either free in the endomysium, within the myofiber sarcoplasm, or within macrophages in myofibers (Figure 1). Electron microscopy revealed microsporidia of the *Anncaliia* genus (Figure 2). We confirmed *A. algerae* by using PCR DNA amplification and sequence analysis.

The patient was started on albendazole (400 mg 2×/d orally) and cyclosporine (100 mg 2x/d orally); tacrolimus and methrotrexate were ceased, and the prednisone dosage was reduced. Within 3 weeks, serum creatine kinase had normalized; muscle tenderness and peripheral edema had been reduced, and power increased. The patient had onset of limb contractures. Because of the ongoing immunosuppression required to manage GVHD, albendazole was continued for ≈9 months. Seven months after the patient’s initial examination, a repeat muscle biopsy indicated no evidence of infection.

## Discussion

Our review of published case reports and patient records indicated that systemic *A. algerae* infection has manifested as a skeletal muscle myositis (Table 1), with central nervous system and cardiac involvement documented in some cases (*1*–*5*). Dysphagia caused by bulbar muscle weakness is a particular concern because it has led to aspiration pneumonia (*2*). Limb contractures have not previously been described and, in the case of our patient, might have been related to GVHD.

**Table 1:** Clinical features of 7 case-patients with *Anncaliia algerae* microsporidial myositis from North America and New South Wales, Australia*

Clinical feature	No. cases
Weakness	7
Muscle pain	7
Fever	6
Fatigue	6
Peripheral edema	6
Weight loss	5
Dysphagia	4
Glossitis	4
Diarrhea	4
Delirium	3
Congestive cardiac failure	1

*In 2 cases the clinical features were only sourced from published reports (*1*,*5*) rather than patient records (*2*–*4*).

Investigation findings in published case reports and patient records are summarized in Table 2 (*1*–*5*; Table 2). Muscle biopsies led to the diagnoses (*1*–*5*). Although Warthin-Starry and Gomori trichrome stains have been optimal for light microscopy, the spores can be confused with yeast cells because of their appearance (*2*,*3*; Figure 1). The features on transmission electron microscopy that allowed identification to the genus level include diplokaryotic nuclei, the absence of a parasitophorous vacuole, vesicotubular appendages, and 8–11 polar tubule coils (*1*–*6*; Figure 2). Species identification has been made with PCR amplification of the small subunit ribosomal RNA gene and sequence analysis by using DNA extracted from muscle and cerebrospinal fluid (*1*–*5*).

**Table 2:** Serologic and laboratory test results for 7 case-patients with *Anncaliia algerae* microsporidial myositis from North America and New South Wales, Australia, 2004*

Test	Abnormal result	No. cases
Serum creatine kinase	Elevated	7
Cardiac troponin	Elevated	2
Erythrocyte sedimentation rate and C-reactive protein	Elevated	5
Full blood count	Lymphocytopenia	6
Serum albumin	Decreased	5
Alanine aminotransferase and aspartate aminotransferase	Elevated	5
Serum creatinine	Elevated	2
Urinary protein	Elevated	3
Nerve conduction studies, electromyography	Myopathy, axonal neuropathy	6
Brain radiologic imaging	Cerebral lesions	2
Cardiac magnetic resonance imaging	Biventricular dysfunction	1
Small subunit rRNA gene PCR, muscle	*A. algerae* DNA	7
Small subunit rRNA gene PCR, cerebrospinal fluid	*A. algerae* DNA	1

*In 2 cases test results were only sourced from published reports (*1*,*5*) rather than patient records (*2*–*4*).

Successful management of *A. algerae* infection requires minimizing immunosuppression, avoiding complications such as aspiration pneumonia, and starting treatment based on albendazole (*2*). A β-tubulin sequence analysis and in vitro assays were consistent with *A. algerae* sensitivity to albendazole, although some viable spores remained in cell cultures after treatment (*7*). In a case of severe illness where substantial immunosuppression and treatment failure of albendazole monotherapy were factors, the addition of fumagillin was effective (*5*). The fumagillin, for which supplies were restricted, was obtained from the manufacturer in France through the Health Canada Special Access Program (*5*). Supply is also restricted in other jurisdictions, including the United States, where an Emergency Investigational New Drug application is required. In the case of the patient we describe, a management strategy was to change the calcineurin inhibitor from tacrolimus to cyclosporine, in light of in vitro evidence that cyclosporine chemosensitized *Encephalitozoon* spp. to the effect of albendazole (*8*).

*A*. *algerae* infects the aquatic stages of mosquitoes when larvae ingest the sports or hatch from contaminated eggs (*9*). Attempts to infect athymic mice by intravenous, oral, and intranasal routes were unsuccessful; however, direct injection of spores into the tail and feet led to infection of myocytes, neural tissue, connective tissue, and bone marrow (*10*). Ingestion, inhalation, and direct inoculation are also possible routes of human infection. A diarrheal illness before hospitalization might indicate a gastrointestinal source, but stool microscopy and gut biopsies have been negative (*2*–*4*). The 2 infected lung transplant recipients described in the literature might have been susceptible to inhaled infection (*3*,*4*). Infection through a mosquito bite is regarded as less likely because the organism has not been found in the saliva of feeding mosquitoes, and exposure to water substantially increased the rate of germination in spores from mosquito tissue (*10*,*11*). Previous patients have resided near sources of environmental water, such as golf courses and woodlands (*2*). The case-patient we describe lived adjacent to a eucalypt forest environment and drank and showered with water from a rainwater tank system that might have contained mosquito larvae or had inflow from water-filled roof gutters containing mosquito larvae. Immunocompromised persons are advised to seek medical guidance before the consumption of rainwater tank water, and until further information regarding transmission is available, other sources of untreated water should be also avoided (*12*).

Clinical case reports lead to a greater understanding about the epidemiology, pathogenesis, and management of *A. algerae* myositis. Considering the widespread use of immunosuppressive therapies and the need to minimize the risk for infection, other priorities for research include the environmental biology of this pathogen and clarification of the transmission route to humans.
